# HIV and disability: a pilot study exploring the use of the Assessment of Motor and Process Skills to measure daily life performance

**DOI:** 10.7448/IAS.16.1.17339

**Published:** 2013-01-21

**Authors:** Brenda Merritt, Jacqueline Gahagan, Anders Kottorp

**Affiliations:** 1 School of Occupational Therapy, Dalhousie University, Halifax, Canada; 2 Health Profession Division, School of Health and Human Performance, Dalhousie University, Halifax, Canada; 3 Department of Neurobiology, Care Sciences and Society, Division of Occupational Therapy, Karolinska Institutet, Huddinge, Sweden

**Keywords:** HIV/AIDS, ADL, AMPS, occupational therapy, health promotion

## Abstract

**Introduction:**

Limitations in performing activities of daily living (ADL) are important indicators of health and overall wellness, yet relatively few studies specifically identify the ADL abilities of people living with HIV/AIDS (PHAs). Given the wide range of abilities and ages of PHAs, there is an urgent need to utilize an assessment of ADL ability that can validly evaluate those who are very able, as well as those who are very disabled, without the presence of ceiling or floor effects, to provide sensitive measures to detect change.

**Purpose:**

The purpose of this study was to gather preliminary evidence of the validity of using the Assessment of Motor and Process Skills (AMPS) with PHAs.

**Methods:**

By utilizing existing data records of PHAs from the international AMPS database, we explored (a) demographic factors; (b) person response validity by examining person and individual item goodness-of-fit to the AMPS measurement model; and (c) trends in ADL abilities of PHAs.

**Findings:**

There were 137 data records in the international AMPS database (0.08% of the database). Goodness-of-fit analyses revealed that >90% of the individuals in the sample fit AMPS measurement model and >99% of the individual item ratings fit the model. More than 80% of the data record had ADL motor abilities that were significantly lower than age expectations, and 67% had ADL process ability measures that were significantly lower than age expectations.

**Conclusions:**

The findings indicate that the AMPS is a valid measure of ADL ability for PHAs. Coupled with the lower than expected number of AMPS data records for PHAs and the significant ADL ability challenges that were encountered by this sample, this pilot study may indicate that PHAs encounter barriers to accessing rehabilitation services and/or may not receive referrals until significant ADL challenges are encountered.

## Introduction

HIV has become characterized as an episodic, chronic health condition in which individuals experience ongoing cycles of health and function as well as illness and disability [1–3]. While people living with HIV/AIDS (PHAs) are living longer as a result of the introduction of antiretroviral (ARV) therapy in the mid-1990s, current evidence indicates that they are at a higher risk of early death and/or disability due to health conditions that are not solely associated with the consequences of HIV/AIDS but may also be related to long-term use of ARVs (e.g. cardiovascular disease, pulmonary disease, hepatic disease, cancer, depression and neurocognitive disorders) [4–7]. Promoting the health and wellness of PHAs through the use of sensitive assessments of disability is an important health promotion consideration, particularly as individuals with HIV are living longer and with greater risks for developing comorbid health conditions.

While higher rates of negative health outcomes have been well documented in the literature, very little research has been conducted to evaluate and measure the everyday functional consequences of living with HIV/AIDS. The International Classification of Functioning, Disability and Health (ICF) [8], developed by the World Health Organization, is an internationally recognized framework that can be used to articulate and differentiate various health and health-related domains/outcomes (i.e. body functions, body structures, activities, participation, environmental factors). When considering the activities and participation domains of the ICF [8], researchers have documented that physicians often overlook the participation or everyday functional challenges that are experienced by their patients who are living with HIV [9]. This finding is not surprising, as the diagnostic markers and body function impairments that are often monitored by physicians cannot be used to reliably predict how effectively a person performs their everyday activities [10–13]. It stands to reason that if the everyday functional challenges that are encountered by PHAs are not identified, then referrals to appropriate health programmes and services (e.g. occupational therapy, physical therapy, self-management, health promotion) may not occur, which could result in even higher risks for adverse health outcomes.

Furthermore, while limitations in performing activities of daily living (ADL) (e.g. self-care, home maintenance, meal preparation) are important determinants of health and overall wellness [14–16], public health reporting agencies rarely monitor or publish ADL outcomes. As a result, occupational therapists, with expertise in evaluating ADL performance, seldom contribute to measuring public health outcomes or developing health promotion policies and programmes [17]. Given this, there is very limited evidence of (a) how chronic health conditions, such has HIV, impact upon a person's ability to participate in necessary and desired daily activities and (b) if health-promoting policies and programmes lead to improved functional abilities. While occupational therapy services have been shown to enhance ADL performance, vocational abilities, and overall quality of life for those with chronic health conditions [18], relatively few studies have been conducted that specifically identify or seek to address the ADL challenges that PHAs may encounter.

In response, strategies for assessing and measuring the everyday functional abilities of PHAs have been developed. However, several challenges have emerged and new research priorities have been documented. For example, Heaton et al. [19] assert that many of the instruments developed to detect the functional deficits of cognitively impaired persons were originally designed for older persons with dementia, and thus they are not often applicable to PHAs who are younger, nor are they sensitive enough to detect the mild or cyclic changes in function that are often experienced among PHAs. To address this need, Heaton et al. [19] created functional, performance-based assessment strategies that could be completed within a laboratory setting. Their findings revealed that tests of everyday function and the presence of depression were the only two significant predictors of “real-world” function. However, ceiling effects were encountered when using the functional assessments, indicating limited sensitivity and insufficient task challenge for persons who are more able.

In order for researchers and clinicians to document and compare the everyday functional consequences of HIV, there is a need to identify sensitive, valid and reliable measures that can be utilized with diagnostically and culturally diverse persons of all ages and ability levels. A leading and internationally recognized assessment of everyday function is the Assessment of Motor and Process Skills (AMPS) [20,21]. The AMPS is a standardized, performance-based, observational assessment that is used to gain information about the quality of an individual's performance of ADL tasks [20,21]. Since the AMPS is used to evaluate the quality of ADL performance (versus body function impairment), it is not diagnosis-specific and can therefore be used to assess individuals 2 years of age or older, regardless of diagnostic profile.

The AMPS was designed as a client-centred ADL assessment where clients are allowed to choose two relevant, familiar and appropriately challenging tasks to perform for the assessment. The AMPS includes over 110 standardized ADL tasks that range in challenge from easy to hard and include personal care tasks (e.g. grooming/hygiene, dressing), home maintenance tasks (vacuuming, ironing, raking leaves), simple meal preparation tasks (e.g. preparing coffee and toast, making simple sandwiches) and more complex meal preparation tasks (e.g. preparing pasta, salad and a beverage; baking a cake; preparing French toast). Given the chronic, episodic nature of HIV, the higher risk of disabling comorbidities and the wide age range among PHAs, an assessment like the AMPS has an enormous advantage because persons can choose tasks that are culturally and personally relevant, age appropriate and of a suitable challenge.

While the AMPS can be utilized to assess the ADL ability of persons who are 2 years of age and older and with any diagnosis, specific studies have not been conducted to evaluate the utility, validity and reliability of using the AMPS specifically with PHAs. Based on studies that have been conducted among persons with relatively mild physical and/or cognitive impairments [22–24], there is evidence that ceiling effects, decreased sensitivity and decreased ecological relevance can be overcome using the AMPS. In a recent study, Verbraak and colleagues [24] found that a group of persons who were reported to have “fully recovered” (i.e. asymptomatic) from a transient ischemic stroke or a non-disabling stroke obtained AMPS ability measures that were below the norms of their healthy, age-matched peers. Using the AMPS as an evaluative measure, researchers were able to accurately identify persons who had very mild functional challenges. While the participants in this study were able to perform ADL tasks independently, the AMPS ability measures accurately indicated that they were not performing at the same level as they used to (i.e. the persons were less efficient and/or demonstrated mild effort and/or clumsiness when completing the tasks), and as a result supplemental intervention strategies were implemented to further enhance their everyday functional abilities. In effect, through using a sensitive measure, greater opportunities were created for early identification of daily life challenges and the implementation of proactive intervention strategies to promote health and daily life function and prevent future ADL decline and/or other adverse health outcomes.

The purpose of this study was to gather preliminary evidence of the validity of using the AMPS with persons who are living with HIV/AIDS. Within this, we address the following research questions: (a) Is the AMPS a valid tool for use with PHAs, as evidenced by acceptable person goodness-of-fit-statistics? (b) Do PHAs, who have been evaluated with the AMPS, demonstrate significantly lower ADL motor and/or ADL process ability measures than their healthy, age-matched peers? and (c) Where sufficient data exist for comparison across groups – are there statistically significant differences in mean ADL motor ability or ADL process ability across different diagnostic groups (e.g. HIV/AIDS as a singular diagnosis, HIV/AIDS+stroke, HIV/AIDS+psychiatric diagnosis)?

## Methods

### Study design and population

This is a retrospective, descriptive and comparative analysis of existing data records from the international AMPS database. This database is stored and maintained by the AMPS Project International. The database houses anonymized data that has been personally submitted by occupational therapists for the purpose of rater calibration and does not typically include data from evaluations that are conducted after rater calibration. The demographic data housed within the database includes geographic region, age, gender, diagnosis and global functional level. All of the demographic categories (except age) are entered into the user's AMPS software through the utilization of drop-down (preset) menu options.

The sample for this study includes pre-existing data records of individuals in the international AMPS database, including men and women from any geographic region, who have been diagnosed with HIV/AIDS. The data records within the international AMPS database are from individuals who were assessed by occupational therapy practitioners within typical occupational therapy settings (e.g. hospitals, client homes, rehabilitation clinics) from a variety of world regions, including both rural and urban areas. Data records selected for the study met the following inclusion criteria: (a) had a primary or secondary diagnosis of HIV/AIDS; (b) were not associated with rater scoring error; (c) had not been utilized for rater education/training; and (d) had known sex, age and global functional level ratings. Prior to initiating this study, ethics approval was obtained from the Dalhousie University Research Ethics Board.

### Instrumentation

As stated previously, the AMPS is a standardized evaluation tool of the quality of occupational performance of ADL tasks [20]. More specifically, 16 ADL motor and 20 ADL process skill items (Table 1) are scored according to the client's observed effort, efficiency, safety and independence when completing familiar and relevant ADL tasks. ADL motor skills are the observable, goal-directed actions that an individual enacts when moving oneself or a task object [20]. For example, when making a pot of coffee, sweeping the floor or putting on socks, one must *reach* for *grip* and *lift* the coffee pot, broom, or socks [20]. ADL process skills are the observable actions of performance that the person enacts to logically sequence the actions, select and use appropriate tools and materials, and adapt performance when problems arise [20]. The AMPS consists of two unidimensional scales of ADL ability, the ADL motor and the ADL process ability scales [20].

**Table 1:** Skill categories and items in the assessment of motor and process skills

Motor skills		Process skills	
*Body position* Stabilizes Aligns Positions *Obtaining and holding objects* Reaches Bends Grips Manipulates Coordinates	*Moving self and objects* Moves Lifts Walks Transports Calibrates Flows *Sustaining performance* Endures Paces	*Sustaining performance* Paces Attends Heeds *Applying knowledge* Chooses Uses Handles Inquires *Temporal organization* Initiates Continues Sequences Terminates	*Organizing space and objects* Searches/Locates Gathers Organizes Restores Navigates *Adapting performance* Notices/Responds Adjusts Accommodates Benefits

From Refs. [20] and [21].

An AMPS evaluation consists of a series of steps, beginning with preparing for the AMPS interview [20]. Within this initial step, from the list of possible ADL tasks, the occupational therapist identifies those tasks that the client knows how to perform, are culturally relevant to the client, can be performed in the test environment and present sufficient challenge to the client [20]. During the AMPS interview, the occupational therapist narrows the list to approximately five ADL tasks; from this shortened list, the client chooses at least two ADL tasks to perform; and he/she decides which task to perform first. Next, the client is familiarized with the testing environment.

Clients are observed while performing ADL tasks and are scored on their performance according to standardized criteria [20]. The client's performance on each ADL motor and ADL process skill is rated on a four-point ordinal scale. A score of “4” indicates the client's observed performance was competent, a score of “3” means the client's observed performance was questionable, a score of “2” indicates the client's performance on that ADL skill item was ineffective and, lastly, a score of “1” means the client's performance was markedly deficient. The occupational therapist then enters the client's scores for each ADL skill item (for each task performed) into his/her password-protected application of the AMPS computer-scoring program [25]. The AMPS computer-scoring program uses a specialized multi-faceted Rasch analysis program [26] to convert raw ordinal ADL motor and ADL process scores into linear interval data of ADL motor and ADL process ability measures. Through the utilization of multi-faceted Rasch analysis, the AMPS computer-scoring program generates linear ADL motor and process ability measures by taking into account the (a) challenge of each of the tasks performed, (b) severity of the rater and (c) difficulty of the ADL motor and ADL process skill items.

### Data analysis

A descriptive analysis (e.g. frequencies) of the demographic factors of the data records was conducted to determine (a) the frequency and type of HIV co-morbidities; (b) geographic region; (c) age; (d) sex; and (f) date of evaluation. Next, to evaluate the validity of using the AMPS with PHAs, the data were subjected to Rasch analysis using the FACETS computer software [25], and the resulting person goodness-of-fit-statistics were analyzed, with a *MnSq* ≤1.4 and associated *z*<2 indicating acceptable goodness-of-fit to the model [20,27]. In general, a 5% rate of misfit among the sample is acceptable (*z <*2) and will not threaten person-response validity [27]. In keeping with the goodness-of-fit analyses of others [27–30], we supplemented our analysis by also evaluating the overall percentage of misfitting ratings, with an acceptable rate of misfit ratings (*t*≥3) set at ≤2.5%.

While the ADL abilities of healthy individuals improve from childhood to adulthood and then begin to naturally decline after the age of 50 [20,31], we do not know if PHAs (with or without a co-morbid condition) follow a similar pattern. To analyze age expectations, the ADL motor and ADL process ability measures of each data record were compared to normative measures [30] and categorized/coded as being (a) within two standard deviations (SD), (b)≥2 SD or (c)≥3 SD from the mean measures of their healthy, age-matched peers. To evaluate if sex, age or diagnostic categories were significantly associated (*p*<0.05) with the above-mentioned normative rankings, a Kruskal-Wallis test of significance was conducted.

Finally, to further evaluate the validity of the generated AMPS ADL motor and process ability measures, we used the established cut-off measures that demarcate the need for assistance to live in the community [20,32,33] to create a new “predicted need for assistance” variable. Using the cut-off measures to guide categorization, each data record was coded as either needing assistance to live in the community or as being independent in the community based upon the generated AMPS motor and process ability measures. This predicted need for assistance was then compared to occupational therapists’ professional judgement of the person's actual need for assistance in the community. Based on Merritt's earlier work [33], when ADL motor and process decisions match (i.e. both measures are either below or above the respective cut-off measures), we expected correct predictions for at least 85% of those who were rated as independent by the evaluating therapist (i.e. sensitivity) and at least 83% for those who were rated as needing assistance (i.e. specificity). When using only the ADL motor ability measure to predict a need for assistance, we expect sensitivity and specificity estimates of 0.67 and 0.72, respectively. When using only the ADL process ability measure to predict need for assistance, we expect sensitivity and specificity estimates of 0.81 and 0.70, respectively [33]. If these expectations are met or exceeded, this would provide an additional line of validity evidence that the AMPS ability measures accurately reflect the therapists’ judgments of the actual day-to-day abilities of PHAs.

## Results

### Demographics

At the time of data extraction, there were a total of 168,877 data records in the international AMPS database that were not used during rater training or indicative of rater scoring error. From this potential sample, 137 data records (0.08%) met the inclusion criteria, in that every person in the database who had a primary or secondary diagnosis of HIV/AIDS met the inclusionary criteria. The data records that met our inclusionary criteria were evaluated between the years 1995 and 2011, and ranged in age from 4 to 79 years (mean=40 years, SD=12.3). There were 88 men (64%) and 49 women from varying world regions (i.e. North America (38%), United Kingdom (30%), Europe (25%), New Zealand/Australia (4%), Asia (1.5%) and Africa (1.5%)). The sample included persons who were reported to be independent in the community (13%), in need of minimal assistance to live in the community (36%) and in need of moderate to maximal assistance to live in the community (51%) (Tables 2 and 3).

**Table 2:** Number of data records by diagnosis and functional level

Diagnostic group	Independent	Minimal assistance	Maximal assistance	Total
HIV/AIDS	4	6	18	28
HIV/AIDS+psychiatric diagnosis	7	14	16	37
HIV/AIDS+cardio-respiratory	0	3	0	3
HIV/AIDS+stroke	1	8	12	21
HIV/AIDS+memory impairment	1	3	3	7
HIV/AIDS+medical diagnosis	1	1	1	3
HIV/AIDS+musculoskeletal impairment	2	3	2	7
HIV/AIDS+neurologic disorder	1	9	18	28
HIV/AIDS+vestibular impairment	0	2	0	2
HIV/AIDS+visual impairment	1	0	0	1
Total	18 (13%)	49 (36%)	70 (51%)	137

**Table 3:** Mean age and ADL ability of PHAs by functional level

	Independent	Minimal assistance	Maximal assistance	Total
Mean age years (SD)	38.4 (11.2)	42 (12.5)	39.7 (13.8)	40.4 (13)
Mean ADL motor (SD)	2.17 (0.70)	1.27 (0.76)	0.70 (0.92)	1.10 (0.97)
Range of ADL motor ability	2.82–3.41	−0.19–3.22	−1.98–2.74	−1.98–3.41
Mean ADL process (SD)	1.62 (0.70)	0.91 (0.42)	0.43 (0.69)	0.76 (0.72)
Range of ADL process ability	0.53–2.82	−0.9–1.87	−1.28–1.72	−1.28–2.82

The data included 28 persons (20%) who had a single diagnosis of HIV/AIDS; the remaining persons in the sample had at least one comorbid health condition (Table 1). The co-morbid diagnoses were coded into the global categories, which included psychiatric illness (e.g. depression, anxiety, schizophrenia, bipolar disease), cardiac and respiratory illnesses, stroke, neurologic disorder (e.g. peripheral nerve damage, multiple sclerosis, brain injury, brain tumour, Parkinson's disease, inflammatory brain disorder), memory impairment, medical diagnosis (e.g. diabetes, high blood pressure), musculoskeletal impairment (e.g. broken bone, hip replacement, torn ligament, osteoarthritis, rheumatoid arthritis), visual impairments and vestibular disorder. Specific diagnostic trends by age and sex were difficult to discern given the relatively small diagnostic groupings; however, the data showed that comorbid health conditions were more common within this sample after the age of 30 years, while the proportion of males and females within each diagnostic grouping was representative of the proportion of males and females within the overall sample.

### Person goodness-of-fit

Of all the persons in the data set, 92% (*n=*126) demonstrated acceptable goodness-of-fit to the ADL motor scale, and 92% demonstrated acceptable goodness-of-fit to the ADL process scale. The data were evaluated further, and no specific trends in the data could explain the reason for the slightly higher than expected proportion of misfitting subjects (e.g. no trends by age, sex, geographic region, age, diagnosis). When evaluating the proportion of misfitting ratings, we expect no more than 2.5% of all of the ratings to misfit, on the motor scale there were 36 misfitting ratings (out of a possible 5033 ratings) and on the process scale there were 42 misfitting ratings (out of a possible 6312 ratings). Thus, <1% of the ratings misfit either the motor or process scales.

### Predicted need for assistance to live in the community

When using the ADL motor cut-off measure of 1.5 logits to predict the need for assistance, 78% of the sample that was originally rated as independent in the community (*n*=18) was correctly classified as being independent (sensitivity=0.78), and 74% of the sample in need of assistance (*n*=119) was correctly classified as needing assistance (specificity=0.74). When using the ADL process cut-off measure of 1.0 logit, sensitivity and specificity estimates were 0.79 and 0.75, respectively. When the ADL motor and process cut-off decision points matched (*n*=88), 82% of the sample (*n*=16) that was originally rated as independent in the community was correctly classified as being independent (sensitivity=0.82), and 90% of the sample in need of assistance (*n*=72) was correctly classified as needing assistance (specificity=0.90).

### Age-expected ADL ability measures

The majority of the persons in the sample (*n*=82, 60%) had ADL motor ability measures that were at least 3 SD below age expectations, with only 20% of the sample obtaining motor ability measures that were within age expectations. With regard to age-expected ADL process ability measures, the distribution across the three different categories was more evenly distributed, 33% of the sample had ADL process ability measures that were within age expectations, 29% were 2 SD below age expectations and 38% of the sample obtained ADL process ability measures that were at least 3 SD below age expectations.

When comparing the age-expected ADL motor and process abilities between men and women, no significant differences were found. When comparing the expected ADL motor and process abilities across age groups, there were no significant differences with regard to ADL process ability. However, there were significant differences with regard to ADL motor ability (*X* ^2^ (5, *N*=137) =13.25, *p*<0.05). Persons under the age of 18 years had a higher probability of meeting ADL motor ability age expectations and those who were older than 41 years of age had a higher probability of being 3 SD below the mean ADL motor age-expected ability measures.

Finally, when comparing the expected ADL abilities across diagnostic groups, there were significant differences with regard to both ADL motor and ADL process ability (*X* ^2^ (9, *N*=137)=22.01, *p*<0.05 and *X* ^2^ (9, *N*=137)=19.9, *p*<0.05, respectively). Further evaluation of the data revealed that PHAs with the comorbid conditions of stroke, other neurologic condition and musculoskeletal impairment were more likely to have ADL motor ability measures that were 3 SD below age expectations. PHAs with the comorbid conditions of stroke, other neurologic condition and memory impairment were more likely to have ADL process ability measures that were 3 SD below age expectations. Finally, PHAs who also had a psychiatric diagnosis were more evenly distributed across the three age expectation categories (i.e. within age expectations, below 2 SD, or below 3 SD age expectations) than the other diagnostic groups.

## Discussion

### Demographics

As we consider the representation of men and women within this dataset, in the United Kingdom and North America between 66% and 74% of the HIV cases are men [34,35], and therefore our dataset is seemingly consistent with these trends. When considering the representation of HIV/AIDS data records within the international AMPS database, we note that in 2009, in spite of approximately 1.5 million adults and children in North America and approximately 86,500 persons in the United Kingdom living with HIV [36], the international AMPS database contains only 52 HIV/AIDS data records from North America and 41 records from the United Kingdom. The number of AMPS data records of persons from these two world regions who have other chronic health conditions is vastly different and provides one potential line of evidence that PHAs may not be accessing occupational therapy services. For example, within the international AMPS database, there are approximately 925 persons from the United Kingdom and North America who have multiple sclerosis (MS; B. Berg, personal communication, October 12, 2011). Given that there are approximately 500,000 persons in the United Kingdom and North America who are living with MS [37–39] and over 2 million who are living with HIV/AIDS, within these two world regions we expected to find more than 93 AMPS data records of PHAs. In support, Worthington and colleagues [40] conducted a Canadian survey of rehabilitation professionals and found that only 39% (*n*=1058) had knowingly ever served an HIV-positive client and 66% reported that they did not believe that they had adequate skills to work with clients who are living with HIV.

Possible explanations for this may be that PHAs do not experience ADL limitations until they are quite unwell and/or hospitalized with an acute condition and therefore do not receive referrals to occupational therapy. Alternatively, healthcare professionals may not be evaluating or screening PHAs for ADL or participation challenges, and as a result the presence of ADL challenges may be overlooked and appropriate referrals to rehabilitation specialists may not occur. The most likely explanation may be that health professionals only refer PHAs to occupational therapy once a *significant* problem is encountered when completing everyday tasks and participating in desired daily life activities. In this case, there is a possibility that many PHAs with mild to moderate ADL challenges may not receive referrals to occupational therapy and/or other health and wellness-focused rehabilitation services. Finally, it is possible that there is a small subgroup of PHAs who demonstrate ADL limitations, and these individuals are sufficiently targeted by occupational therapy services. While our findings raise many questions that deserve further exploration and critical analysis, it is our belief that there are societal and organizational barriers that prevent PHAs from accessing and receiving the rehabilitation services that are needed to promote safe and effective ADL performance, decrease the need for assistance, prevent decline in ADL abilities and/or prevent the emergence of comorbid health conditions.

### Validity (goodness-of-fit) and predicted need for assistance

Despite such concerns, the data have illuminated several important implications. First, the person goodness-of-fit statistics and proportion of misfitting ratings supports the claim that the AMPS generated valid measures for more than 90% of the HIV data records. When evaluating the proportion of misfitting ratings, we found that<1% of the ratings misfit. This finding met our criteria of<2.5% misfit, and thus we suggest that the generated AMPS motor and process ability measures were valid to use for further statistical analysis.

Given the diagnostic heterogeneity of the sample, the results correspond well with earlier studies with people who have complex diagnostic profiles [41]. Future studies with larger and more distinct subsamples may generate knowledge about unique performance profiles that may (a) explain the slightly higher degree of person misfit in this sample and (b) generate detailed information about strengths and weaknesses in everyday/ADL performance that may support the development of health promotion and/or chronic disease self-management programmes.

The ADL ability measures of this sample of PHAs appear to accurately reflect the therapists’ professional judgment of the day-to-day abilities that promote or hinder independent living in the community. More specifically, we found that the predicted need for assistance (based on ADL motor and process abilities) accurately reflected the evaluating therapists’ evaluation of the need for assistance in the community. Our estimates of sensitivity and specificity were overall consistent with Merritt's earlier work [33]. More specifically, all of the estimates of specificity found within this study were slightly higher than those documented by Merritt [33]. When using ADL motor ability as the predictor, our estimates of sensitivity were higher than Merritt's, 0.78 versus 0.67. However, when using ADL process ability as the predictor or when using matched motor/process decisions, our estimates for sensitivity were slightly lower than those documented by Merritt [33]. The minor discrepancies in our empirical findings and Merritt's original work may be related to the small and disproportional sample sizes in the subgroups.

When investigating the slightly lower estimates of sensitivity, additional analyses revealed that three of the data records demonstrated inconsistent scoring between the therapists’ rating of overall functional level and problems demonstrated during the AMPS evaluations; these results may be reflective of rater scoring error (i.e. too strict) or reflective of incomplete information provided by the PHA to the therapist regarding the amount of assistance needed for community living or the challenges encountered during daily task performances (i.e. the PHA may not have fully divulged their daily life challenges). Regardless, our overall findings provide one line of evidence to support that the AMPS is a valid measure of ADL ability among PHAs and may also provide evidence to support a PHA's likely need for assistance to live in the community. Through utilizing the AMPS, healthcare providers may be able to effectively identify daily life challenges and subsequently develop proactive intervention strategies to promote health, daily life function and to prevent future ADL decline and/or other adverse health outcomes.

In contrast to other studies [19], it is important to note that we did not encounter any ceiling effects (i.e. ADL motor ability measures did not exceed 4.00 logits, and ADL process ability measures did not exceed 3.00 logits) (Table 2). Since the AMPS includes more than 110 standardized tasks that range from easy to hard, includes both personal and instrumental ADL tasks that can be performed within and outside of the home, we believe that there is greater opportunity for the client to perform tasks that are culturally relevant and present an appropriate level of challenge. In effect, this diminishes the opportunity for ceiling effects and testing error. These findings also support the sensitivity of the AMPS to detect changes in ADL abilities of PHAs that are due to treatment strategies, disease progression and/or episodic illness.

### Age-expected ADL ability

The PHAs within this study demonstrated ADL limitations to an extent where assistance is likely needed to manage living in the community. Alarmingly, 80% of the sample had ADL motor ability measures that were more than 2 SD below their healthy, age matched peers, and 67% of the sample had ADL process ability measures that were more than 2 SD below their healthy, age matched peers. While these figures cannot equate to the ADL abilities of all PHAs, it may be a fair representation of those who have been referred to occupational therapy services. This may indicate that PHAs do not receive referrals to occupational therapy until significant challenges are encountered when performing everyday tasks. Therefore, there exists a need to critically explore not only the ADL abilities of a representative sample of PHAs but also the potential systemic and social barriers that may limit access to rehabilitation services.

When comparing the ADL ability measures of PHAs to normative (expected) ability measures, men and women demonstrated similar trends. However, we found that ADL motor limitations were more pronounced with increased age; those who were greater than 40 years of age had a higher likelihood of obtaining ADL motor ability measures that were 3 SD below their healthy peers. Similarly, PHAs with comorbid conditions of stroke and other neurologic conditions were more likely to have ADL motor and ADL process ability measures that were at least 3 SD below their healthy, age-matched peers. PHAs with musculoskeletal and memory impairments were more likely to obtain ADL motor or ADL process ability measures that were at least 3 SD below their healthy, age-matched peers, respectively. Others have found that PHAs who have HIV-associated neurocognitive disorders often have accompanying motor limitations [42]. Through utilizing one assessment, we were able to document everyday challenges that were related to physical effort (ADL motor ability), as well as inefficiency (ADL process ability).

The above findings were not entirely surprising given that others have documented accelerated aging among PHAs [5], which could result in accelerated rates of functional decline. This further illustrates the need to enhance access to rehabilitation services across the age continuum to promote health, safety and effective ADL performance and/or prevent the emergence of comorbid health conditions. Ideally, functional/ADL screening should be a standard part of the HIV continuum of care, especially after the age of 30 years.

### Limitations

One limitation of this study is that it is unknown if the sample evaluated are representative of all PHAs. For example, due to the episodic nature of HIV and the lack of diagnostic specific information available from the database, there is no way of knowing the point along the continuum of disability that is represented by this sample and what impact this could have on our estimations of the validity of the AMPS for use with PHAs. In addition, due to the relatively small sample size, we were not able to directly compare the validity (i.e. person goodness-of-fit) between men and women. While we did not identify any consistent trends across the misfitting ratings related to sex, additional studies may be warranted. Another limitation is the sample size and the limited demographic information (e.g. ARV status, time since HIV diagnosis, etc.). Further empirical research using the AMPS with PHAs should be performed using a longitudinal design to study the consequences over time onto ADL ability. Regardless of these limitations, this study offers a preliminary exploration of the ADL abilities of PHAs; our findings contribute to the discussion of the potential functional consequences of living with HIV/AIDS and indicate the need for further studies focusing on evaluating ADL ability with sensitive assessments that demonstrate evidence of validity for the specific target group.

## Conclusions

This study provides preliminary evidence that the AMPS can be used as a valid assessment of ADL abilities among PHAs, as it demonstrates (a) evidence of person response validity in a sample with PHAs; (b) sensitivity in the measures generated, demonstrated in the absence of ceiling and floor effects; and (c) validity of the ADL motor and process ability measures in relation to overall professional judgment of the need for support to function in community. Furthermore, the empirical findings of the study highlight that PHAs may demonstrate ADL limitations that fall well below age expectations in both ADL motor and ADL process abilities, with the risk of functional decline increasing with age. As one of the goals for health-focused interventions aimed at PHAs is promoting health and overall wellness across the lifespan, our findings indicate that future HIV research should focus not only on ADL functioning across the lifespan but also equitable access to rehabilitation services [16].
